# Effect of Bioactive Compound of *Aronia melanocarpa* on Cardiovascular System in Experimental Hypertension

**DOI:** 10.1155/2017/8156594

**Published:** 2017-11-30

**Authors:** Martina Cebova, Jana Klimentova, Pavol Janega, Olga Pechanova

**Affiliations:** ^1^Institute of Normal and Pathological Physiology, Slovak Academy of Sciences, Bratislava, Slovakia; ^2^Department of Pathology, Faculty of Medicine, Comenius University, Bratislava, Slovakia

## Abstract

*Aronia melanocarpa* has attracted scientific interest due to its dense contents of different polyphenols. We aimed to analyse effects of *Aronia melanocarpa* (AME) extract on blood pressure (BP), lipid peroxidation, cytokine level, total NOS activity in the left ventricle (LV), and aorta of L-NAME-induced hypertensive rats. 12-week-old male WKY rats were assigned to the control group and groups treated with AME extract (57.90 mg/kg/day), L-NAME (40 mg/kg/day), or combination of L-NAME (40 mg/kg/day) and AME (57.90 mg/kg/day) in tap water for 3 weeks. NOS activity, eNOS protein expression, and conjugated diene (CD) concentration were determined in the LV and aorta. After 3 weeks of L-NAME treatment, BP was increased by 28% and concomitant treatment with AME reduced it by 21%. NOS activity of the LV and aorta in the L-NAME group was decreased by about 40%, while AME increased it almost on the control level. AME-induced eNOS upregulation may contribute to increase NOS activity. Moreover, AME decreased CD concentration in the LV and aorta and TNF-*α* and IL-6 production in the plasma were increased by L-NAME treatment. In conclusion, our results showed that active substances of *Aronia melanocarpa* may have a positive effect on blood pressure, NOS activity, and proinflammatory processes in L-NAME-induced hypertension.

## 1. Introduction

Hypertension is a cardiovascular risk factor associated with endothelial dysfunction and oxidative stress as well. This can lead to a reduction of NO availability and vasodilatation. Those processes participate in increasing systemic blood pressure and myocardial remodelling and in the development of cardiac hypertrophy [1, 2]. Inhibition of nitric oxide synthase by N^G^-nitro-L-arginine methyl ester (L-NAME) is a well-established rat model of experimental hypertension with increased blood pressure and contractility in different parts of the vasculature, attenuated vascular relaxation, and decreased heart rate [3, 4]. The structural changes after long-term NO synthase inhibition in the cardiovascular system included left ventricle hypertrophy, remodelling of coronary arteries and aorta, as well as extensive areas of fibrosis and necrosis [4–6]. In hypertension, the increased production of oxygen-free radicals is a generally accepted fact. Usually, there is a balance between the antioxidants and the prooxidants *in vivo*, but several factors like stress, radiation, and nutrition may lead to the so-called oxidative stress, which imposes the necessity to contribute exogenous antioxidants with the diet. The injury caused by oxidative stress can affect all organ systems. Therefore, many studies are oriented on antioxidant treatment that may prevent the hypertension and associated organ alterations.

Increasing consumption of polyphenol-rich foods is a promising strategy to reduce cardiovascular risk. Increased flavonoid consumption correlated with positive effect on low-density lipoproteins, blood pressure, and flow-mediated dilation [7]. Polyphenols with numerous biological activities are used for their ability to act as an antioxidant either by scavenging reactive oxygen species (ROS) or inhibiting enzymes involved in the ROS production [1, 8, 9].

*Aronia melanocarpa*, also known as black chokeberry, belongs to the Rosaceae family, originally coming from North America. Nowadays, it is commonly used also in Europe due to a high content of nutrients being beneficial for the health. The components of the aronia are dependent on many factors such as cultivar, fertilization, maturation of the berries, harvest date, or habitat/location [10, 11]. The most abundant components are anthocyanins and flavonoids [12]. Chokeberries with high resistance to frost and mechanized harvesting are widely used in processed and derived products including juices, wines, jellies, and tea [13, 14]. The protective role of the main components from aronia against cardiovascular diseases came up from clinical trials [15, 16] and animal studies [17–19]. Attention has been also focused on chokeberries due to their antioxidant properties related to the high polyphenolic content [20, 21].

The aim of our study was to determine the effect of nonalcohol *Aronia melanocarpa* extract on NO synthase activity, particularly NO synthase isoform protein expressions in the aorta and left ventricle, as well as their immunohistochemistry and on cytokine and conjugated diene levels on L-NAME-induced experimental model of arterial hypertension.

## 2. Materials and Methods

### 2.1. Chemicals

Most of the chemicals and reagents were obtained from Sigma-Aldrich; when not, the company is indicated.

### 2.2. *Aronia melanocarpa* Extract

Samples of *Aronia melanocarpa* wine (Winery Pereg Ltd.) were subjected to the process of dealcoholisation and concentration, producing an alcohol-free *Aronia melanocarpa* extract (AME) (previously described by Kondrashov [22]). The total phenolic content of AME was assessed according to the Folin-Ciocalteu method. Briefly, 1 mL of AME, 1 mL of Folin-Ciocalteu's reagent, and 5 mL of distilled water were mixed together. The solution was incubated for 5 min at a room temperature in the darkness. Then, 1 mL of 20% Na_2_CO_3_ was added. The solution was made up to 10 mL, mixed, and incubated for 1 h at a room temperature in the darkness. The absorbance of AME sample was measured at 765 nm against a blank (corresponding extraction mixture was used instead of algal extract) on UV/VIS spectrometer Lambda 25 (PerkinElmer, Waltham, MA, USA). Gallic acid was used as a standard to construct the calibration curve (20, 40, 60, 80, and 100 mg·L^−1^). The total phenolic content of AME is expressed in mg·g^−1^ of gallic acid equivalent (GAE).

### 2.3. Animal Study

#### 2.3.1. Animals and Treatment

All procedures and experimental protocols were approved by the Ethical Committee of the Institute of Normal and Pathological Physiology SAS and conform to the European Convention on Animal Protection and Guidelines on Research Animal Use.

12-week-old male Wistar Kyoto rats were divided into the control group, the group treated with AME in the dose 57.90 mg/kg/day, the group treated with N^G^-nitro-L-arginine methyl ester (L-NAME) in the dose 40 mg/kg/day, and the group treated with L-NAME in the dose 40 mg/kg/day + AME 57.90 mg/kg/day. Each group obtained 6 animals. L-NAME and AME were administered *via* the drinking water from the 12th week of age for 3 weeks. Daily water consumption was estimated individually for every animal and adjusted, if necessary. All animals were housed at a temperature of 22–24°C and fed with a regular pellet diet *ad libitum*. Blood pressure (BP) was measured noninvasively, using tail-cuff plethysmography weekly. At the end of treatment, the animals were sacrificed; body weight (BW) and heart weight (HW) were determined. The HW/BW ratio was calculated. Samples of the left ventricle and aorta were used to determine NO synthase activity, eNOS and iNOS protein expressions (Western blot and immunohistochemistry), and conjugated diene level. Cytokine levels were measured in the plasma.

#### 2.3.2. Total NOS Activity and Protein Expression

Total NO synthase activity was determined in crude homogenates of the left ventricle and aorta by measuring the formation of [^3^H]-L-citrulline from [^3^H]-L-arginine (ARC, Montana, USA) as previously described and slightly modified by Pechanova [23]. [^3^H]-L-citrulline was measured with the Quanta Smart TriCarb Liquid Scintillation Analyzer (Packard Instrument Company, Meriden, CT).

Protein expressions of eNOS and iNOS were determined in the aorta and left ventricle by Western blot analysis. The samples were probed with polyclonal rabbit, anti-eNOS, anti-iNOS, and anti-GAPDH antibodies (Abcam, Cambridge, UK). The intensity of bands was visualized using the enhanced chemiluminescence system (ECL, Amersham, UK), quantified by using ChemiDoc™ Touch Imagine System (Image Lab™ Touch software, Bio-Rad), and normalized to GAPDH bands.

#### 2.3.3. Cytokine Level and CD Determination

Cytokine levels were determined by Bio-Plex Pro™ rat cytokine, chemokine, and growth factor assays in plasma.

The concentration of conjugated dienes (CD) was measured in lipid extracts of the left ventricle homogenates [24]. After chloroform evaporation under inert atmosphere and addition of cyclohexane, conjugated diene concentrations were determined spectrophotometrically (*λ* = 233 nm, GBC 911A, Bio-Rad Laboratories).

#### 2.3.4. Immunohistochemical Analysis of eNOS and iNOS in LV

The tissue samples were processed in a standard manner, embedded in paraffin, and sectioned; 3 *μ*m thick slices were deparaffinized and rehydrated in phosphate-buffered physiological saline solution (10 mM, pH 7.2). The tissue epitopes were damasked using the automated water bath heating process in Dako PT Link (Agilent, Santa Clara, California); the slides were incubated in citrate retrieval solution (10 mM citrate, pH 6.0) at 98°C for 20 minutes. The slides were subsequently incubated 2 hours at room temperature with the primary mouse monoclonal IgG2a antibody against eNOS or iNOS (Santa Cruz Biotechnology, Dallas, USA, sc-376751) diluted 1 : 100. The samples were immunostained using anti-mouse anti-rabbit immune-peroxidase polymer (Histofine, Nichirei Biosciences, Tokyo, Japan) for 30 minutes at a room temperature according to the manufacturer's instructions. For visualization, the diaminobenzidine substrate-chromogen solution was used (DAB, Agilent, Santa Clara, California) for 5 minutes. The slides were counterstained with hematoxylin. The DAB positivity was evaluated by light microscopy and measured by histomorphometry using the Fiji morphometric software [25] based on ImageJ 1.51n platform [26]. The final results are expressed as proportional ratio comparing to the mean DAB positivity of evaluated marker in WKY controls (expressed as 1.00).

### 2.4. Statistics

The results are expressed as mean ± SEM. One-way analysis of variance and Duncan test were used for statistical analysis. Values were considered significant with a probability value *p* < 0.05.

## 3. Results

### 3.1. AME Content

The total content of nonalcohol extract of *Aronia melanocarpa* is shown in Table 1. The total phenolic content adjusted on gallic acid equivalent was 57,870 mg/L.

The most predominant mineral elements detected in AME were ferrum (Fe), zinc (Zn), and cuprum (Cu). The results are visualized in Table 2.

### 3.2. Animal Studies

#### 3.2.1. Cardiovascular Parameters and Plasma Cytokine Level

After three weeks of L-NAME treatment, BP was increased by 28% in comparison to the control group. Concomitant treatment by AME reduced BP by 21% (Table 3). At the end of the experiment, HW/BW ratio (mg/g) was 2.72 ± 0.05 in the control group. This ratio was increased in L-NAME group (2.95 ± 0.10) versus control rats. AME was able to decrease this value on the control level (2.77 ± 0.02) (Table 3).

#### 3.2.2. Cytokine and CD Concentration

**Figure 1:**
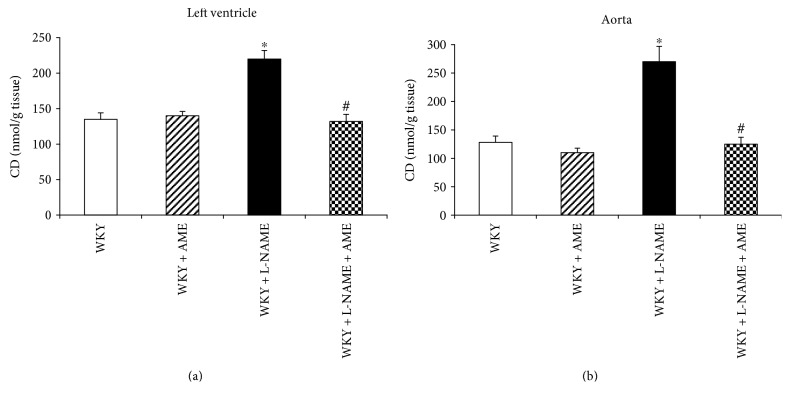
Conjugate diene (CD) level of the left ventricle (a) and aorta (b) of Wistar Kyoto rats treated with AME (57.90 mg/kg/day), with L-NAME (40 mg/kg/day), and with L-NAME (40 mg/kg/day) + AME (57.90 mg/kg/day) for 3 weeks and age-matched controls. Data are means ± SEM, significant differences: ^∗^*P* < 0.05 compared to age-matched controls; ^#^*P* < 0.05 compared to L-NAME group.

AME was able to inhibit TNF*α*, and IL-6 production increased in the L-NAME group (Table 3). L-NAME administration increased the level of CD in both investigated tissues. Furthermore, AME was able to decrease the level of CD almost on the level of controls (Figures 1(a) and 1(b)).

#### 3.2.3. Total NO Synthase Activity and Protein Expression

**Figure 2:**
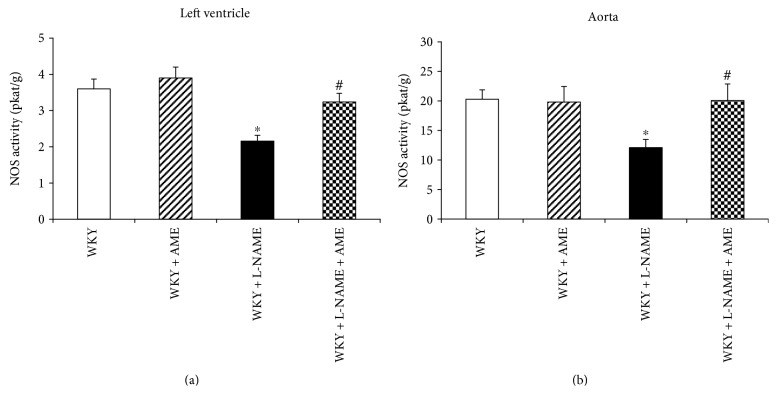
Nitric oxide synthase (NOS) activity of the left ventricle (a) and aorta (b) of Wistar Kyoto rats treated with AME (57.90 mg/kg/day), with L-NAME (40 mg/kg/day), and with L-NAME (40 mg/kg/day) + AME (57.90 mg/kg/day) for 3 weeks and age-matched controls. Data are means ± SEM, significant differences: ^∗^*P* < 0.05 compared to age-matched controls; ^#^*P* < 0.05 compared to L-NAME group.

The total NO synthase activity was decreased after 3 weeks of L-NAME treatment in both the left ventricle and aorta. However, AME was able to increase NO synthase activity in the left ventricle on 90% of the control value (Figure 2(a)) and on control value in the aorta (Figure 2(b)).

**Figure 3:**
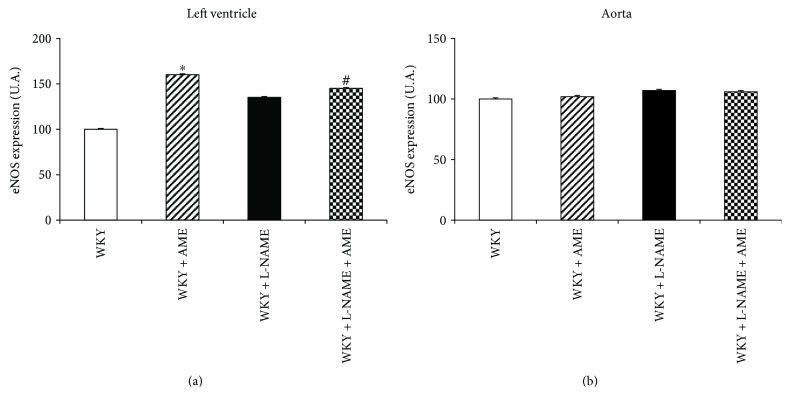
Endothelial NOS (eNOS) expression of the left ventricle (a) and aorta (b) of Wistar Kyoto rats treated with AME (57.90 mg/kg/day), with L-NAME (40 mg/kg/day), and with L-NAME (40 mg/kg/day) + AME (57.90 mg/kg/day) for 3 weeks and age-matched controls. Data are means ± SEM, significant differences: ^∗^*P* < 0.05 compared to age-matched controls; ^#^*P* < 0.05 compared to L-NAME group.

Endothelial NOS protein expression was upregulated only in LV of AME group (Figure 3). The L-NAME administration alone and concomitant treatment with AME had the tendency to increase eNOS expression as well. We did not observe any changes of eNOS expression in the aorta.

Inducible NOS protein expression was not changed in any investigated tissue and group (data are not shown).

#### 3.2.4. Immunohistochemical Analysis of eNOS and iNOS in LV

**Figure 4:**
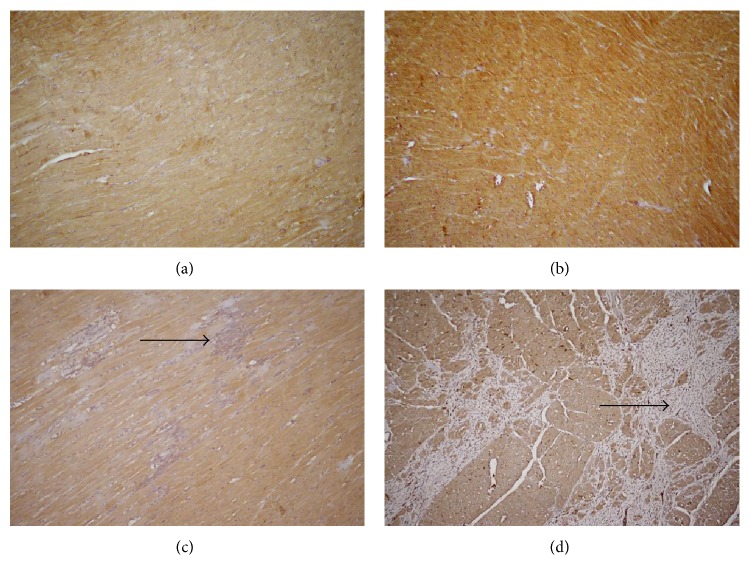
eNOS expression in heart tissue. Diffuse cytoplasmic positivity of eNOS was found in all samples. Only the AME treatment without the L-NAME administration significantly increased the eNOS expression. (a) WKY, (b) AME, (c) L-NAME, (d) L-NAME + AME, eNOS, Ab-Poly, DAB, 200x.

The eNOS showed diffuse and regular cytoplasmic positivity in cardiomyocytes of all experimental animals. Only the AME treatment without the L-NAME administration significantly increased the eNOS expression (Figures 4(a)–4(d)). Positivity of eNOS is well correlated with its protein expression analysed by Western blot. Similarly, as Western blot, immunohistochemistry did not show any changes in iNOS protein expression between the groups (data are not shown).

## 4. Discussion

Long-lasting inhibition of NO synthase induces hypertension, endothelial and contractile dysfunction, inflammation, and fibrosis enlargement [27]. Chronic inhibition of NO synthase by L-NAME causes increase of blood pressure associated with vascular structural changes [4, 28]. It has been also shown that L-NAME increased fibrosis and left ventricular hypertrophy [3, 29]. Thus, L-NAME-induced hypertension represents a useful tool for studying increase of blood pressure associated with inflammatory processes and fibrosis. Lowering blood pressure is one of the most important means to reduce cardiovascular morbidity and mortality. Our study showed that *Aronia melanocarpa* extract resulted in significant reduction of blood pressure in L-NAME-treated rats that may be due to high polyphenolic content. In our previous study, we have shown that polyphenols can act as antioxidants, due to their functional groups and aromatic structure [30]. Our finding is in agreement with Ciocoiu [31] in experimental hypertension in rats. They found reduction of systolic and diastolic pressures after treatment with *Aronia melanocarpa* extract. Other authors showed decreased blood pressure in SHR after the commercial *Aronia melanocarpa* extract *Aronox* treatment [32]. The hypotensive effect was revealed also by Naruszewicz [33] in patients after myocardial infarction with statin therapy and by Tjelle [34] in hypertensive volunteers.

Several studies showed that hypertension may lead to overproduction of free radicals, which could be the reason of biochemical, molecular, and behavioural changes [35, 36]. In this study, concentration of conjugated dienes (CD) was determined as a marker of oxidative damage and lipid peroxidation. Our previous results observed increased production of free radicals in the L-NAME model of hypertension [37]. We confirmed increased concentration of CD in the aorta and left ventricle after L-NAME treatment. AME extract was able to decrease the CD on the control level. Similarly, improved plasma and hepatic antioxidant function were found in apo E^−/−^ mice after chokeberry extract treatment [38]. In the same pattern, IL-6 and TNF- *α* levels were decreased in the hypertensive group treated with AME. Han and Nicholson [39] showed significant drop of IL-6 as well as reduction in the level of oxidative stress in cardiac patients after chokeberry treatment. Nowadays, chokeberry consumption is in good relation with human immune system. Mechanisms of action are mediated by inhibition of cytokine IL-6, IL-8, and TNF-*α* in human monocytes as well as by activation of NF*κ*B and prostaglandin E_2_ [40].

The antioxidant activity of *Aronia melanocarpa* is mostly contributed by the phenolic compounds [40] and depends on the structure of polyphenol functional groups [41]. It is also known from the literature that an increased level of free radicals leads to uncoupling of the NOS dimer to a monomer form, and an activity of this enzyme as well as production of NO is decreased. Moreover, under conditions of oxidative stress, NO synthase synthesizes rather superoxide radical than NO [37, 42]. This, besides direct L-NAME inhibition, may be one of the reasons of decreased NO synthase activity in our experiment. AME treatment in our experiment directly increased NO synthase activity probably by both decreasing oxidative stress by polyphenolic group function and stabilizing NO synthase dimer by addition of Zn.

This study provides also the evidence that active compounds of AME are likely to stimulate the expression of eNOS in myocardium; however, in combination with L- NAME, this stimulation is not significant. Those results are in good agreement with Wallerath [43] who found increased eNOS expression in humans after polyphenol treatment. The positive effect on endothelial formation of NO in coronary arteries was observed also by Kim [38]. Authors showed that phosphorylation of eNOS via redox-sensitive activation of Src/PI3/Akt pathway is mostly by conjugated cyanidins and chlorogenic acids, which are also the most, represented content of our extract.

## 5. Conclusions

In conclusion, AME treatment was able to reduce blood pressure in L-NAME-induced hypertension by decreasing oxidative stress and increasing NO production. Both direct contribution of polyphenolic groups and Zn addition may be responsible for NO synthase upregulation in LV. Moreover, in the same tissue, AME alone was able to increase eNOS protein expression and had a tendency to increase it during the concomitant treatment with L-NAME which may further contribute to NO synthase activity increase.

## Figures and Tables

**Table 1:** Components of *Aronia melanocarpa* extract (in *μ*g/g).

Component	(*μ*g·g^−1^)
Gallic acid	2.24 ± 0.01
Protocatechuic acid	340.86 ± 7.70
4-Hydroxybenzoic acid	21.44 ± 0.31
Epigallocatechin	354.81 ± 11.19
Catechin	149.68 ± 1.91
Chlorogenic acid	1948.60 ± 0.59
Vanillic acid	14.61 ± 0.11
Caffeic acid	4.78 ± 0.09
Syringic acid	4.32 ± 0.29
Epicatechin	29.24 ± 0.52
Trans-p-coum acid	19.37 ± 1.32
Ferulic acid	173.90 ± 1.94
Ellagic acid	42.78 ± 0.14
Rutin	17.44 ± 0.00
T-2-hydroxycinnamic acid	1.18 ± 0.12
Protocatechuic acid etylester	11.55 ± 0.39
Resveratrol	16.65 ± 0.32
Cinnamic acid	2.35 ± 0.00
Kaempferol	23.09 ± 0.79
Quercetin	0.00 ± 0.00

Data are expressed as mean values (*n* = 3) ±SD.

**Table 2:** Elemental profile of *Aronia melanocarpa* extract (in ng/mL).

Sample	K	Mg	P	Fe	Cu	Pb	Zn	Na
AME	12.12 ± 0.09	85.41 ± 0.82	14.47 ± 0.12	1960.71 ± 11.05	405.14 ± 2.87	28.62 ± 2.15	950.34 ± 4.35	12.19 ± 0.13

Data are expressed as mean values (*n* = 3) ± SD.

**Table 3:** Blood pressure (BP), relative heart weight (HW/BW), TNF*α* (pg/mL), and IL-6 level (pg/mL) of Wistar Kyoto rats treated with AME (57.90 mg/kg/day), with L-NAME (40 mg/kg/day), and with L-NAME (40 mg/kg/day) + AME (57.90 mg/kg/day) for 3 weeks and age-matched controls.

Group	BP (mmHg)	Hw/Bw (mg/g)	TNF*α* (pg/mL)	IL-6 (pg/mL)
WKY	118.39 ± 4.29	2.72 ± 0.05	12.11 ± 2.41	23.41 ± 3.78
WKY + AME	122.04 ± 1.81	2.55 ± 0.05	7.32 ± 2.12	22.14 ± 5.22
WKY + L-NAME	155.11 ± 3.71^∗^	2.95 ± 0.10^∗^	18.19 ± 1.38^∗^	36.43 ± 3.08^∗^
WKY + L-NAME + AME	129.22 ± 2.51	2.77 ± 0.02^#^	13.89 ± 1.29^#^	25.13 ± 2.11^#^

Data are means ± SEM, significant differences: ^∗^*P* < 0.05 compared to age-matched controls; ^#^*P* < 0.05 compared to L-NAME group.
